# Simultaneous Subtotal Gastrectomy and Right Colectomy for Synchronous Gastric and Colon Cancer: A Case Report

**DOI:** 10.7759/cureus.3892

**Published:** 2019-01-15

**Authors:** Raul Mederos, Jose R Lamas, Anika Ramos, Ayesha Farooq, Syeda K Farooq

**Affiliations:** 1 Surgery, Hialeah Hospital, Hialeah, USA; 2 Surgery, Aga Khan University, Karachi, PAK; 3 Surgery, Liaquat National Medical College, Karachi, PAK

**Keywords:** synchronous, gastric cancer, colon cancer

## Abstract

Synchronous gastric and colon cancer although reported from East Asia (China, Japan, Korea) remain rare in other parts of the world. We present the case of a 50-year-old lady who presented to the Hialeah Hospital, USA with an eight-month history of generalized abdominal pain and upon investigation was found to have dual gastric and colonic malignancy. While the incidence of gastric cancer has dropped drastically in the USA, colon cancer remains the third most frequent cancer in both men and women. An estimated 2%-17% of oncological patients may be affected by multiple primary malignancies and a high degree of clinical suspicion along with appropriate diagnostic procedures is required for a definitive diagnosis.

## Introduction

The advent of sophisticated treatments in the field of cancer has led to increased survival times among oncological patients. In 2014, more than 14 million Americans were living with a history of cancer [1]. Patients with a history of cancer have a higher risk of developing multiple primary neoplasms, i.e. more than one synchronous or metachronous tumor in the same or different organs [2]. Widely reported in China, Japan, and Korea, we present the case study of a dual primary malignancy involving stomach and colon from the USA, where such cases are relatively uncommon.

## Case presentation

A 50-year-old Hispanic lady presented with an eight-month history of dull, aching generalized abdominal pain that worsened after meals, and was associated with nausea and reduced appetite. Her stool had become softer than before, but there was no melena, and no hematochezia. She had episodes of severe abdominal pain that disrupted her sleep every few days. Review of systems revealed no weight loss. Her past medical history was significant for hypertension, and past surgical history revealed the following four surgeries within the last 10 years: laparoscopic cholecystectomy, laparoscopic appendectomy, umbilical hernia repair, and tubal ligation. She had been a lifelong nonsmoker and had never used alcohol. Her family history revealed breast cancer in her mother and prostate cancer in her father. Her BMI was 38. Physical examination was unremarkable. She underwent an upper gastrointestinal endoscopy that showed a nonobstructing, nonbleeding cratered ulcer in the posterior wall of the gastric body. Biopsies were taken that revealed moderate-poorly differentiated adenocarcinoma. She underwent positron-emission tomography (PET) scanning that showed increased uptake in two regions: one in the medial gastric fundus (maximum SUV=14.2) and a second one near the cecum (maximum SUV=18.6). Further the PET scan showed abnormal circumferential thickening and pericolonic inflammatory changes involving the cecum, and numerous small lymph nodes were noted in the right lower quadrant (largest lymph node=12 mm × 18 mm). One week later, she underwent a colonoscopy that showed a fungating, partially obstructing, nonbleeding, circumferential mass in the cecum. Biopsy showed a moderately differentiated adenocarcinoma. The blood tests showed anemia (hemoglobin= 9.3) while all other tests were unremarkable. Carcinoembryonic antigen (CEA) was 0.819 preoperatively. Based on the above investigations it was decided that the patient has synchronous gastric and colon cancer and that it is surgically resectable. A week later, she underwent an exploratory laparotomy, subtotal gastrectomy with Roux-en-Y reconstruction and right colectomy with resection of terminal ileum and ileocolostomy. The description of the procedure is as follows: in the supine position on the OR table after being appropriately identified, induction of anesthesia, endotracheal tube placement, area of the abdomen prepped, and draped in the standard sterile fashion. A midline laparotomy incision was made from the xiphoid down to the umbilicus. That incision was carried down from the skin and subcutaneous tissue until the abdomen was entered. An Alexis wound retractor was placed for better exposure. Internal organs were grossly unremarkable. Liver was inspected and noted to be free of any metastatic lesions. There were two malignant appearing tumors, one in the body of the stomach and another one in the cecum. Both tumors were easily identified by palpation although both were tattooed. We commenced the operation with the gastric portion. We created an opening to the lesser sac and transected the branches of the gastroduodenal artery along the greater curvature of the stomach. Then we excised the branches of the right gastric artery supplying the lesser curvature. The tumor itself was located in the body of the stomach that would save the junction between the proximal third and the middle third of the stomach. So we fired a thoracoabdominal (TA-90) stapler with a few centimeters margin into the proximal stomach. Then we proceeded by firing a gastrointestinal anastomosis (GIA-75) stapler along the pylorus, transecting approximately two-thirds of the stomach. The specimen was opened, and we decided to perform a Roux-en-Y reconstruction. We went a couple of feet from the ligament of Treitz and a GIA stapler was used to separate the jejunum. The jejunum was brought retrocolic all the way up into the proximal stomach. To create an anastomosis we chose an end-to-end anastomosis (EEA-25) stapler. The anvil was placed inside the proximal stomach creating an enterotomy that was approximated using another fire on the GIA stapler. Then we proceeded by making an opening in the small bowl and through that opening the EEA-25 stapler was then carefully introduced and it was opened. It was then connected to the anvil previously placed in the proximal stomach under direct visualization; the stapler was approximated and fired. The stapler was removed showing two donuts indicating a patent anastomosis. The opening where the stapler entered the small bowel was sealed using an Echelon stapler. Then we proceeded by curetting the anastomosis between the two limbs of the jejunum. It was a side-to-side anastomosis using GIA and TA. Anastomosis reinforced using 3-0 Vicryl sutures and noted to be widely patent with good coloration and tension free. Anesthesia proceeded by advancing a nasogastric tube all the way down to the anastomosis. Evicell was sprayed in the area to ensure complete hemostasis. 

We then concentrated our attention on the right lower quadrant where the colon was visualized. The tumor was in the right colon near the cecum. We started mobilizing the colon medial to the white line of Toldt ensuring hemostasis. The proximal transverse colon was also mobilized by transecting the hepatocolic ligaments. We proceeded then by firing a GIA in the terminal ileum; another GIA was fired at the proximal transverse colon. Then using the Echelon stapler, series of fires were used to transect the right mesocolon. We then proceeded by anastomosing the terminal ileum and the transverse colon. It was side-to-side functional end-to-end anastomosis using GIA and TA. Anastomosis reinforced using 3-0 Vicryl and it was noted to be widely patent with good coloration and tension free. 

The abdomen was then irrigated with saline. The fluid was aspirated. No evidence of bleeding or injury to intra-abdominal organs was noted. The Alexis retractor was removed. The fascia was approximated using a running PDS, and the skin was approximated using staples followed by sterile dressing applied on top. The patient tolerated the procedure well without any complications. She was then transferred to recovery in stable condition. The counts were correct ×2.

**Figure 1:**
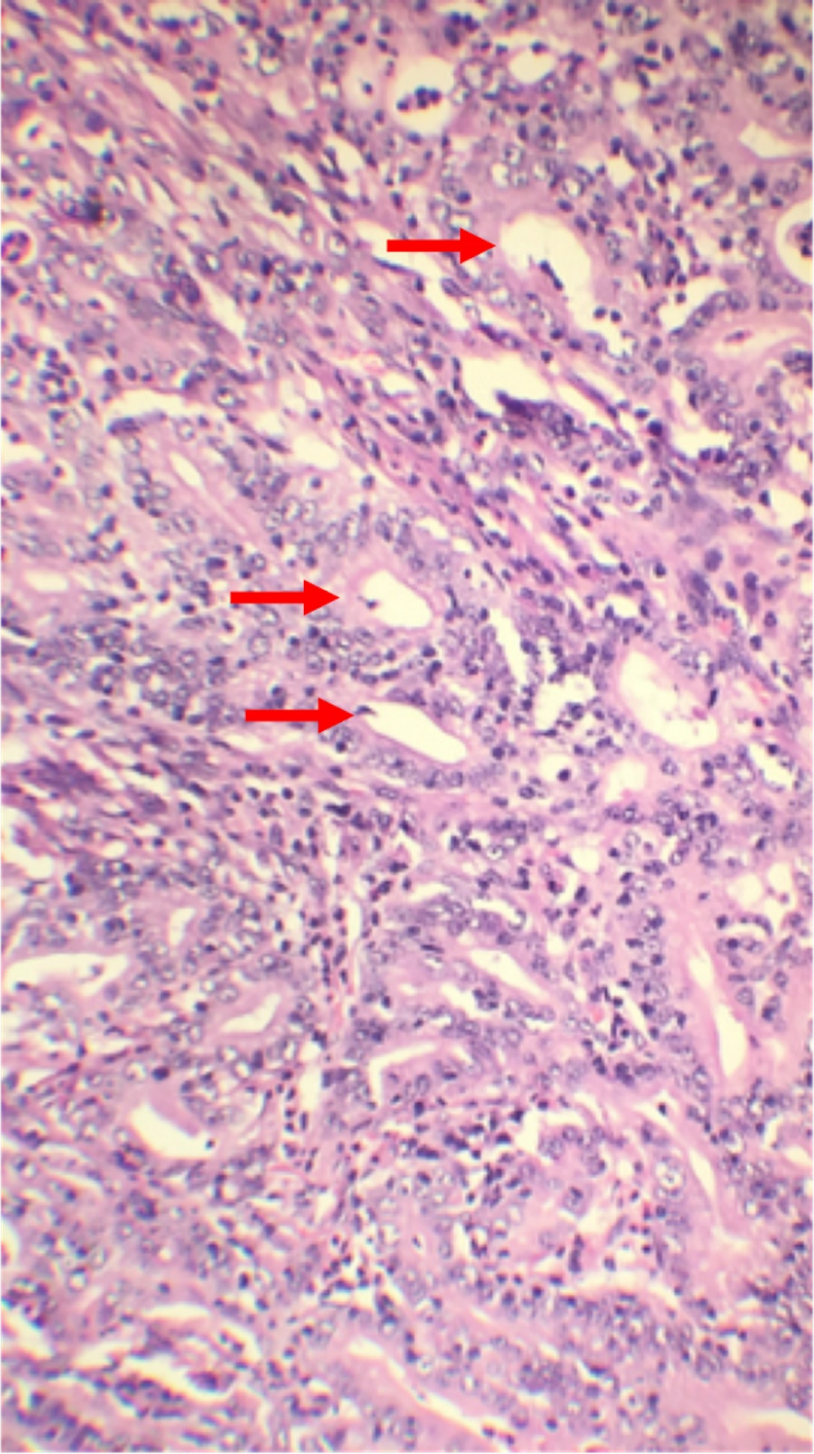
Histopathology of distal stomach showing poorly differentiated adenocarcinoma of the intestinal type.

Both surgical specimens were sent to pathology. The surgical margins of both masses and the 21 excised nodes were tumor free. The distal gastrectomy specimen showed poorly differentiated adenocarcinoma of the intestinal type (pT2aN0Mx) (Figure 1).

**Figure 2:**
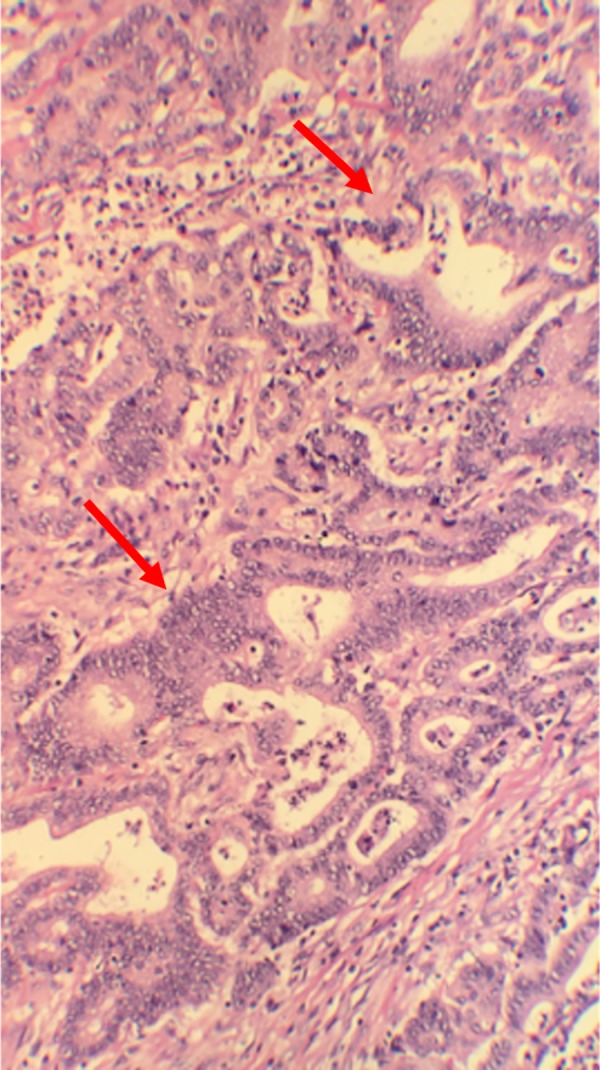
Histopathology of colon showing moderately differentiated adenocarcinoma.

**Figure 3:**
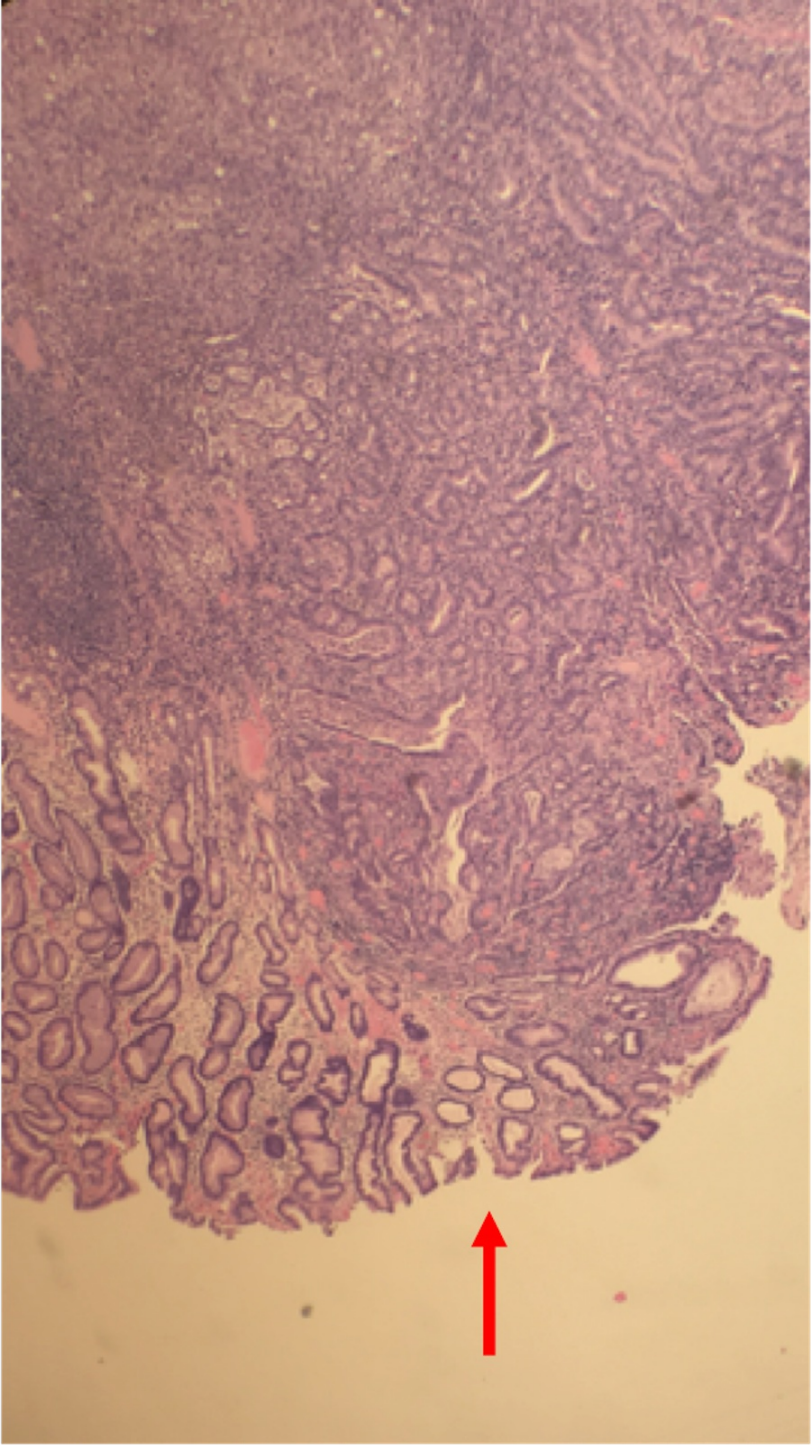
Histopathology slide of colon showing cancer cells invading muscularis propria and extending into the serosa.

The right colectomy specimen showed moderately differentiated adenocarcinoma that was invading the muscularis propria and extending into the subserosa (pT2aN0Mx) (Figures 2-3). 

Postoperatively the patient remained well (she was ambulating, tolerating oral diet, and pain was under control) and was discharged on the fourth postoperative day. She followed up in clinic up to two months after the surgery. She was about to begin chemotherapy. She missed her latest clinic appointment.

## Discussion

We report a case of concurrent gastric and colon cancer in a middle-aged lady from the USA. Colon and gastric cancer are two of the five most common malignancies worldwide, along with lung, breast, and prostate cancer. While, three East Asian (China, Japan, Korea) countries account for more than 60% of the global burden of gastric cancer, the incidence of gastric cancer in the USA has fallen by 80% since 1950 [3]. Colorectal cancer, however, is the second leading cause of cancer-related deaths in the USA and third most common cancer in both sexes [4]. 

In 1932, Warren and Gates proposed the first definition of multiple primary neoplasms stating that: (a) each tumor must be malignant by histology; (b) each tumor must be anatomically distinct; and (c) the second tumor should not be a recurrence or metastasis of the first one. A synchronous cancer is defined as any cancer that occurs within six months of the first one whereas a metachronous cancer is one that occurs at least six months after the first cancer [2]. Metachronous tumors are more common than synchronous tumors. Second primary tumors are more common than third or fourth primary tumors [5]. In the literature the incidence of multiple primary neoplasms ranges from 2% to 17% [1].

With advances in diagnostic modalities, there has been a surge in the incidence of multiple primary neoplasms. Patients with gastric cancer, for instance, are at an increased risk of developing a secondary neoplasm [2]. Further, the advent of novel chemotherapeutic drugs has led to increased survival rates among cancer patients. Cancer survivors have a 20% greater risk for developing a second primary malignancy than the general population [5].

Genetic, environmental, and lifestyle factors act synergistically in the pathogenesis of multiple primary neoplasms. The *CHEK2* gene, for instance, has been implicated in concurrent stomach and colon cancer among others such as breast, prostate, thyroid, and kidney. Other examples of gene mutations involved in the pathogenesis of multiple primary neoplasms include the cell-cycle regulator p53 (Li Fraumeni syndrome) and the DNA repair protein BRCA (breast, ovary, and pancreatic cancer). Lifestyle influences such as the use of alcohol, tobacco, and nitrosamines can play a role in cancer pathogenesis as they are known to contain carcinogens [1]. Patients with history of multiple cancers should undergo a complete evaluation including family history, genetic counseling, and cancer screening, to estimate their risk of developing another neoplasm. Women with colonic cancer who have a family history of endometrial or ovarian malignancy, may have Lynch syndrome. Lynch syndrome is caused by an autosomal dominant defect in the mismatch repair proteins leading to microsatellite instability and can cause colon cancer despite the absence of polypoidal growth [1]. The recommended screening modalities for colon cancer and gastric cancer are, respectively, colonoscopy and esophagogastroduodenoscopy. The caveat in diagnosing multiple primary neoplasms is that one cancer can be mistaken for a metastasis of the other one. Hence, it is necessary to biopsy all neoplasms to establish the diagnosis [6].

Family members of patients must be screened. According to the American Cancer Society guidelines for colorectal cancer, for patients younger than 60 at the time of diagnosis (as is the case with our patient) first-degree relatives must begin screening colonoscopy either at age 40 or 10 years before the youngest case in the family was diagnosed. Screening colonoscopy is to be repeated every 5 years [7]. In contrast, routine endoscopic screening for gastric cancer is not recommended because there is evidence that screening in low incidence regions, such as the USA does not lead to a reduction in mortality [8]. Screening endoscopy for gastric cancer is recommended only in high-risk areas such as Japan. Yaghoobi et al. recommend that even in low-risk regions, people who are at high-risk for gastric cancer should be screened and treated for *Helicobacter pylori* infection [9].

## Conclusions

The presence of multiple neoplasms must be considered among all oncological patients who have more than one tumor. Multiple primary neoplasms pose a diagnostic challenge as they can be mistaken for metastasis. A multitude of diagnostic tests, such as endoscopy and radiological imaging can be employed to screen patients and family members, whereas a definitive diagnosis can be established only after biopsy of any suspicious lesions. The treatment should be planned such that it targets all the primary neoplasms.
